# Oxygen-Releasing Hyaluronic Acid-Based Dispersion with Controlled Oxygen Delivery for Enhanced Periodontal Tissue Engineering

**DOI:** 10.3390/ijms24065936

**Published:** 2023-03-21

**Authors:** Lena Katharina Müller-Heupt, Nadine Wiesmann-Imilowski, Sofia Schröder, Jonathan Groß, Pablo Cores Ziskoven, Philipp Bani, Peer Wolfgang Kämmerer, Eik Schiegnitz, Anja Eckelt, John Eckelt, Ulrike Ritz, Till Opatz, Bilal Al-Nawas, Christopher V. Synatschke, James Deschner

**Affiliations:** 1 Department of Oral and Maxillofacial Surgery, University Medical Center Mainz, Augustusplatz 2, 55131 Mainz, Germany; 2 Department of Otorhinolaryngology, University Medical Center Mainz, Langenbeck Str. 1, 55131 Mainz, Germany; 3 Department of Chemistry, Johannes Gutenberg-University, Duesbergweg 10-14, 55128 Mainz, Germany; 4 Department of Periodontology and Operative Dentistry, University Medical Center Mainz, Augustusplatz 2, 55131 Mainz, Germany; 5 WEE-Solve GmbH, Auf der Burg 6, 55130 Mainz, Germany; 6 Department of Orthopaedics and Traumatology, University Medical Center Mainz, Langenbeckstr. 1, 55131 Mainz, Germany; 7 Max Planck Institute for Polymer Research, Ackermannweg 10, 55128 Mainz, Germany

**Keywords:** angiogenesis, biopolymer, hyaluronic acid, hypoxia, tissue regeneration, tissue engineering, oxygen, peroxides, periodontitis

## Abstract

Periodontitis is a chronic biofilm-associated inflammatory disease of the tooth-supporting tissues that causes tooth loss. It is strongly associated with anaerobic bacterial colonization and represents a substantial global health burden. Due to a local hypoxic environment, tissue regeneration is impaired. Oxygen therapy has shown promising results as a potential treatment of periodontitis, but so far, local oxygen delivery remains a key technical challenge. An oxygen (O_2_)-releasing hyaluronic acid (HA)-based dispersion with a controlled oxygen delivery was developed. Cell viability of primary human fibroblasts, osteoblasts, and HUVECs was demonstrated, and biocompatibility was tested using a chorioallantoic membrane assay (CAM assay). Suppression of anaerobic growth of *Porphyromonas gingivalis* was shown using the broth microdilution assay. In vitro assays showed that the O_2_-releasing HA was not cytotoxic towards human primary fibroblasts, osteoblasts, and HUVECs. In vivo, angiogenesis was enhanced in a CAM assay, although not to a statistically significant degree. Growth of *P. gingivalis* was inhibited by CaO_2_ concentrations higher than 256 mg/L. Taken together, the results of this study demonstrate the biocompatibility and selective antimicrobial activity against *P. gingivalis* for the developed O_2_-releasing HA-based dispersion and the potential of O_2_-releasing biomaterials for periodontal tissue regeneration.

## 1. Introduction

Periodontitis, a biofilm-associated inflammatory disease of the periodontal tissues [1,2], is one of the most prevalent global diseases. Indirect costs related to periodontitis in the United States and Europe were estimated to exceed EUR 300 billion in 2018 [3]. Periodontitis is strongly associated with anaerobic bacterial colonization and represents a substantial global health burden due to its epidemiologic associations with other chronic inflammation-driven diseases such as cardiovascular disorders and diabetes [4,5].

Although periodontitis can be successfully treated, restoration of the original form, structure, and function of the periodontal tissues lost due to the disease, i.e., periodontal regeneration, is possible only in certain cases. In addition, the results after periodontal regenerative therapy are only partially predictable. This shows that the treatment of periodontitis is still a challenge.

Due to the chronic inflammation, the vasculature in the periodontal tissues is severely impaired by periodontitis, resulting in a hypoxic microenvironment [6]. On the other hand, the hypoxic milieu within periodontal pockets seems to play a critical role in the disruption of the host–immune hemostasis, periodontal tissue remodeling, and inflammation of periodontal tissues [7,8,9], and is furthermore coupled with overgrowth of anaerobic subgingival microorganisms [10].

At sites where a chronic inflammatory response could be detected, oxygen consumption is increased, and blood flow is stimulated. This change in the partial pressure of oxygen (pO_2_) in the affected tissue is due in part to increased oxygen (O_2_) consumption, including oxygen consumption by resident cells and infiltrated defence cells, and in part to decreased oxygen availability due to endothelial damage and vasoconstrictive microcirculation, as well as facultative anaerobic bacteria. Local hypoxia in periodontitis, in turn, favors the survival of anaerobic Gram-negative pathogens and further lowers the oxygen partial pressure in the environment. Tissue hypoxia in periodontal disease is characterized by an increase in hypoxia-inducible factor 1-alpha (HIF-1α) protein levels, which is detectable in tissue biopsies affected by periodontitis. A hypoxic environment can upregulate the expression of proinflammatory cytokines and matrix metalloproteinases (MMPs) by host cells during periodontal disease [11].

To positively influence the dysbiotic oral microbiota, there are various approaches such as probiotics, parabiotics, and postbiotics as an adjunct to nonsurgical periodontal therapy treatment provided as toothpastes or lozenges, which seem to be beneficial to suppress specific periodontopathogens [12,13,14,15,16]. Another therapeutic strategy involves the use of oxygen.

The oxygen content in healthy periodontal tissue ranges from 2.9% to 5.7% [17], but many causes can lead to local hypoxia of the microenvironment in periodontal tissues, such as severe periodontitis [7]. The oxygen content in periodontal tissues with a mean pocket depth of 6.9 mm is 1.8% [18]. Oxygen is involved in various biological processes such as cell metabolism and signal transduction. In particular, in the wound healing process, the transient oxidative stress induced by oxygen is beneficial for increasing cellular activities, secretion of growth factors, and promotion of neovascularization. Periodontal ligament stem cells (PDLSCs) possess multipotent, highly proliferative, and self-renewing capacities. Furthermore, they exhibit the ability to differentiate into cementoblasts or osteoblasts [19]. The periodontal ligament (PDL), a connective tissue between bone and teeth, composed of multiple cells, such as fibroblasts, PDLSCs, and osteoblasts, provides an oxygen-enriched ecologic microenvironment crucial for healthy periodontal tissues and normal cell functions [20]. Thus, the regeneration of the PDL is a crucial factor for the regeneration of the other periodontal structures also damaged by periodontitis. [21,22,23]. However, it has been shown that the growth rate of PDL fibroblasts in in vitro studies decreases with the reduction of oxygen levels. Moreover, PDL fibroblasts were found to migrate significantly faster—at 21% and 5%, rather than at 1% O_2_ [17]; in addition, a reduction of pO_2_ from 20% to 2% decreased the formation of bone nodules, while it almost disappeared at 0.2% pO_2_ [24].

For the abovementioned reasons—in addition to its ability to inhibit the growth of anaerobic bacteria—hyperbaric oxygen therapy (HBOT) has been tested as adjuvant treatment of subgingival instrumentation for periodontitis, with promising results regarding the reduction of anaerobic bacteria and periodontal parameters [25,26,27]. Nevertheless, HBOT is a time-consuming and expensive procedure. Therefore, topical oxygen therapies may be an interesting therapeutic option to use in dental practice.

For the development of a local O_2_-releasing therapeutic agent for periodontal tissues, the use of a hyaluronic acid (HA)-based biopolymer matrix appeared to be particularly interesting in the case of periodontal adjuvant treatment, since HA is biocompatible, biodegradable, and has wound-healing properties. HA is a biological molecule found in different tissues of the human body. It occurs naturally in the gingiva, periodontal ligaments, dental cementum, and alveolar bone, and unstimulated saliva concentrations range from 148 to 1270 ng/mg [26,27]. Furthermore, HA is an important component of the extracellular matrix and plays an important role in cell migration and proliferation, contributing to wound healing and tissue regeneration [28]. The concentration of hyaluronic acid is tissue-dependent, and its properties are determined by its molecular weight.

In general, high molecular weight (MW) HA (MW > 10^6^ Da) has immunosuppressive and antiangiogenic properties, intermediate size HA (MW from 2 × 10^4^ to 10^6^ Da) positively influences wound healing and regeneration, and small HA molecules (MW from 6 × 10^3^ Da to 2 × 10^4^ Da) contribute to proinflammatory and angiogenic processes. HA is one of the local substances used in the past decade as an adjuvant to nonsurgical periodontal treatment, and most of the HA-based gels used in periodontal therapy contain high-MW HA [27]. It has been reported that high MW HA products do not prolong inflammatory processes, impair the healing process, or induce excessive metalloproteinase (MMP) expression at the repair site in gingival tissue [27]. Other studies found that high MW hyaluronic acid increased the proliferation of human periodontal ligament (PDL) cells and maintained their high viability [29]. Clinical studies in patients with periodontitis have shown a decrease in the proliferation index of gingival epithelium (expression of the Ki-67 antigen) and of the inflammatory process, and improved periodontal lesions [28], bleeding on probing [29,30], the sulcus fluid flow rate [31], plaque index, pocket probing depth [32,33], and clinical attachment level [30,33,34].

Oxygen-releasing biomaterials have been developed with a primary focus on application in tissue engineering, i.e., in large 3D tissue constructs, to overcome insufficient oxygen supply due to a lack of vascularization [35,36,37,38]. Typically, inorganic compounds, such as percarbonates and peroxides, provide a source for oxygen through a chemical decomposition reaction, but fluorinated compounds that can dissolve molecular oxygen have also been reported. Various polymers such as polycaprolactone [39], poly(lactic-co-glycolic acid) [40], and polydimethylsiloxane were used to encapsulate oxygen-producing materials, where their hydrophobic nature improves long-term release of oxygen, necessary for tissue regeneration. There are very few examples of oxygen-releasing materials outside of tissue engineering applications. To the best of our knowledge, there have been no previous reports on the use of such materials to control the growth of anaerobic bacteria.

For the abovementioned reasons, the goal of our research was to develop a biocompatible and biodegradable O_2_-releasing HA-based dispersion suitable for topical periodontitis therapy. To avoid excessive and rapid oxygen generation (burst release), which may lead to oxidative stress and the production of reactive oxygen species (ROS—key signaling molecules for the progression of tissue inflammation and endothelial dysfunction) [41], we aimed to develop a biomaterial with slow and sustained oxygen release.

## 2. Results

### 2.1. Oxygen Release Kinetics of the O_2_-Releasing HA-Based Dispersion

**Figure 1:**
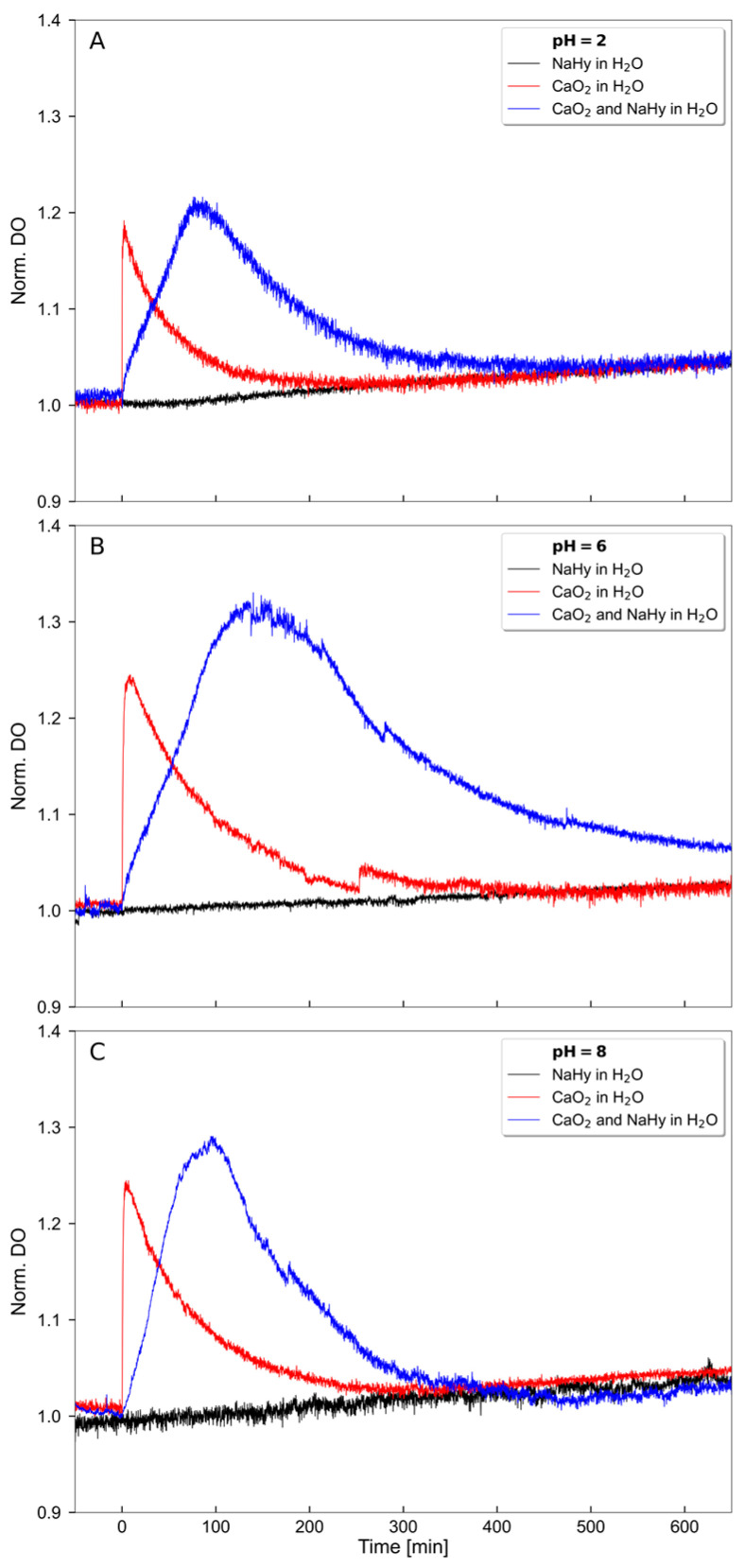
Sustained O_2_ release of CaO_2_ enclosed in an HA-based matrix compared to CaO_2_ or HA only. The experiments were conducted at different pH levels. (**A**) pH 2, (**B**) pH 6, and (**C**) pH 8 with either 50 mg CaO_2_ dissolved in 5 mL aqua (H_2_O) or 5 mL HA matrix compared to baseline over a time period of 700 min. Baseline: 5 mL HA matrix in 0.9 wt% NaCl at 34.1 °C.

We prepared an oxygen-releasing HA material by first dispersing CaO_2_ particles in MilliQ water and slowly adding high MW HA (MW = 1.5–2.5 MDa) under continuous stirring to form a viscous slurry. The ratio between HA and CaO_2_ was optimized to produce a macroscopically homogeneous mixture. The oxygen-release behavior was then evaluated. Delayed oxygen release and an increased amount of available oxygen, as indicated by a larger area under the curve (AUC), was observed when CaO_2_ was enclosed in an HA matrix at different pH levels (Table 1, Figure 1A–C). The greatest AUC, of 98.52, was observed for CaO_2_ and HA in H_2_O at pH 6 (Table 1).

The oxygen release was increased as compared to the release of oxygen from CaO_2_ in H_2_O only. This increase was observed at all pH levels (2, 6, and 8), which is important for the therapeutic use of the dispersion because the pH can vary at different periodontal pocket sites due to inflammation and bacterial metabolites. The release was particularly enhanced at pH 6. Furthermore, the O_2_ release time was increased up to 3.6-fold when CaO_2_ was enclosed in an HA matrix (Figure 1B) compared to free CaO_2_ in solution, which showed a much more rapid burst release when used as single substance without an HA matrix.

### 2.2. Detection of Calcium Peroxide Particles in HA-CaO_2_ Dispersions

**Figure 2:**
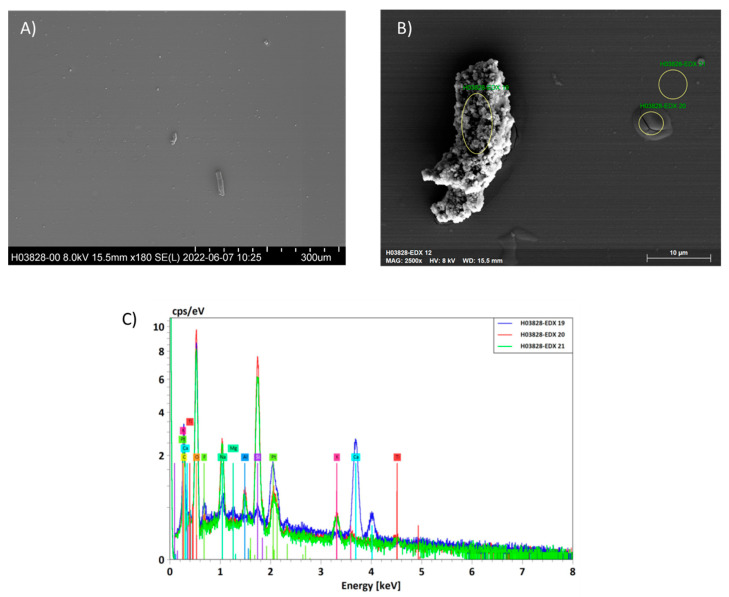
SEM and EDX analysis of drop-cast HA-based dispersion containing CaO_2_. (**A**) Micrograph showing dispersed particles after material deposition. Scale bar represents 300 µm. (**B**) Enlarged image of a single particle with highlighted regions used for EDX analysis shown in (**C**). Scale bar is 10 µm.

To confirm the presence of CaO_2_ particles in the HA-based dispersion, scanning electron microscopy (SEM) and energy dispersive X-ray analysis (EDX) were performed on dried samples of the material (Figure 2). Particles with a diverse size range (appr. 4–30 µm) could be observed. EDX spectra (Figure 2C) confirmed the presence of calcium ions in these particles but not in the surrounding matrix, indicating that the CaO_2_ particles were dispersed in the polymer, but did not fully dissolve.

### 2.3. FTIR of the O_2_-Releasing HA-Based Dispersion

Next, the stability of the HA-based dispersion was investigated by FTIR spectroscopy. When HA is oxidized by strong oxidizing agents (e.g., NaIO_4_), a C–C bond in a glucose unit breaks, and two aldehyde groups are formed. The presence of the latter can be qualitatively proven by a characteristic vibrational excitation band at 1735 cm^−1^ in the IR spectrum. To prove that HA is stable towards oxidation in the dispersion, a freshly prepared sample was compared with a two-month-old sample as well as a seven-month-old sample (each stored at ambient temperature in the absence of light). None of the samples exhibited this characteristic vibration band (Supplementary Materials, Figure S1), indicating that the HA is not degraded during storage.

### 2.4. Biocompatibility of the O_2_-Releasing HA-Based Dispersion

**Figure 3:**
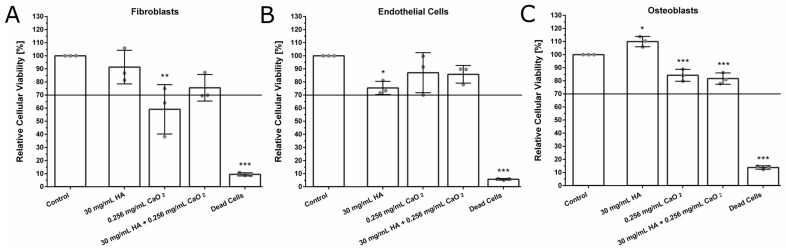
Effect of the O_2_-releasing HA-based dispersion on cellular viability of human primary fibroblasts, endothelial cells, and human primary osteoblasts treated with HA, CaO_2_, or a combination of CaO_2_ and HA. In accordance with ISO 10993-5, cell viability under 70% was regarded as cytotoxicity. Two-way ANOVA. * *p* < 0.05, ** *p* < 0.01, *** *p* < 0.001. (**A**) Fibroblasts treated with 0.256 mg/mL CaO_2_ showed a reduction in cell viability by more than 30%, indicating the cytotoxicity of CaO_2_ as a single substance. N = 3. (**B**) HA showed a slight decrease in cellular viability of endothelial cells, whereas it was slightly increased by CaO_2_ or a combination of CaO_2_ and HA compared to treatment with HA alone. N = 3. (**C**) Osteoblasts hardly showed a reduction of their viability after treatment in comparison to untreated osteoblasts (=100% cell viability). This indicates that both substances, as single substances and in combination, have no cytotoxic effect on osteoblasts. Nevertheless, HA slightly increased cellular viability in comparison to untreated osteoblasts. N = 3.

Since the main goal of our study was to develop a dispersion suitable for the treatment of periodontitis, the biocompatibility of the O_2_-releasing dispersion is a key challenge due to the highly reactive nature of CaO_2_. Therefore, we investigated the effect of the O_2_-releasing HA-based dispersion on the cellular viability of human primary fibroblasts obtained from human oral mucosa, human primary osteoblasts, and human primary umbilical vein endothelial cells (HUVECs), since biomaterials incorporating CaO_2_ may negatively affect cell viability. All cells are crucial for periodontal regeneration. In this context, all cells were treated with HA, CaO_2,_ or a combination of CaO_2_ and HA to examine their cytotoxicity. In accordance with ISO 10993-5, cell viability under 70% was regarded as cytotoxicity. Neither HA alone nor in combination with CaO_2_ was cytotoxic to human primary fibroblasts obtained from different patients (Figure 3A). In contrast, fibroblasts treated with 0.256 mg/mL CaO_2_ showed a reduction in cell viability by more than 30%, indicating the cytotoxicity of CaO_2_ as single substance. Furthermore, none of the substances were cytotoxic for endothelial cells at the tested concentrations, but HA slightly decreased their cell viability (Figure 3B). Finally, none of the substances were cytotoxic for osteoblasts; however, both CaO_2_ and the combination of CaO_2_ and HA slightly decreased cell viability (Figure 3C) compared to HA alone. In contrast, the addition of HA as a single substance increased cell viability compared to the control group.

**Figure 4:**
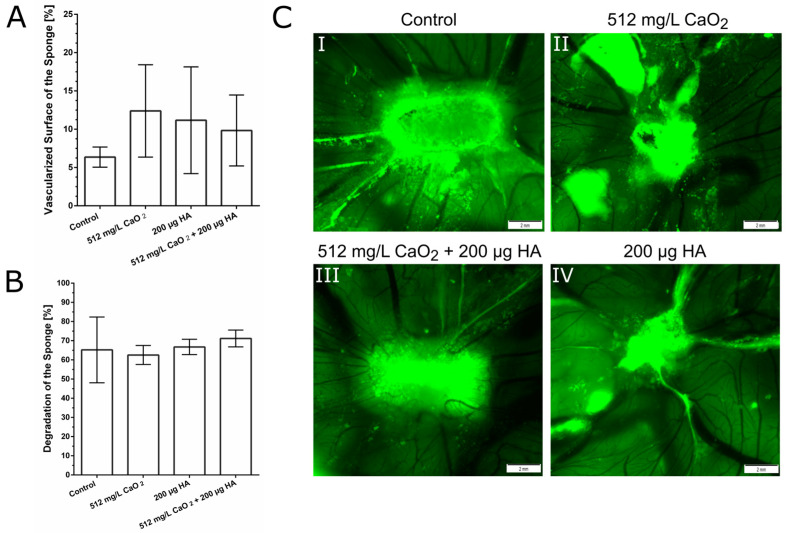
Effect of the O_2_-releasing HA-based dispersion on the CAM. (**A**) Angiogenesis was higher if tissue was treated with HA, CaO_2_, or a combination thereof compared to untreated CAM tissue. The results were not significant. (**B**) None of the tested substances had a cytotoxic effect on the CAM. The degradation of the sponge on which the different substances were applied was not different from the degradation of an empty sponge and no adverse tissue reactions to the substances were seen on the CAM. (**C**) Microscopic images of the CAM tissue treated with either the sponge only (**I**; control), 512 mg/L CaO_2_ (**II**), 512 mg/L CaO_2_ + 200 μg HA (**III**), or 200 μg HA only (**IV**).

Furthermore, biocompatibility and capacity to induce angiogenesis of the O_2_-releasing material were investigated using a chorioallantoic membrane assay (CAM assay). The vascularized tissue surface area, measurable as vascularized surface of the sponge, increased using HA, CaO_2_, or a combination thereof compared to untreated CAM tissue (Figure 4A). Nevertheless, the results were not significant due to high standard deviations. Furthermore, none of the tested substances had a cytotoxic effect on the CAM. The degradation of the sponge to which the different substances were applied did not differ from that of an empty sponge, and no adverse tissue reactions to the substances were observed on the CAM (Figure 4B).

### 2.5. Broth Microdilution Assay

The minimum inhibitory concentration (MIC) for *P. gingivalis* was determined using the broth microdilution assay. The MIC for CaO_2_ dispersions amounted to 256 mg/L.

## 3. Discussion

The aim of this study was to develop a biocompatible and resorbable O_2_-releasing biomaterial for the therapy of periodontitis, which results in tissue hypoxia and consecutively impaired tissue regeneration due to chronic low oxygen supply. In brief, our study revealed that a novel combination of CaO_2_ enclosed into an HA matrix released more oxygen, as indicated by a larger area under the curve, with a sustained release rate, compared to pure CaO_2_, which showed a rapid and high oxygen burst.

Materials with similar properties, but used for purposes other than periodontal therapy, have been described in the literature [35]. In general, similar materials are targeted for use in tissue regeneration, especially in large-tissue models that are vascularly undersupplied and therefore hypoxic. This typically requires a low-threshold release of oxygen, ideally lasting for weeks to months. Approaches used so far to achieve a long-lasting oxygen release are the incorporation of inorganic peroxides such as CaO_2_ into mostly hydrophobic polymers such as polycaprolactone [42], poly(lactide-co-glycolide) [43], human keratin, silk, or gelatin [44]. To enable tissue growth on these materials, the polymers are spun, for example, into nanofibers, which provide a large surface area. While such hydrophobic polymers are well suited for growing cells in culture media, it is often difficult to adapt the materials to complex and small-defect structures, such as those found in periodontal pockets, because the materials are usually not injectable and are difficult to deform.

HA can be oxidatively degraded by adding sodium metaperiodate [45]. Oxidized HA has been reported to contain multiple aldehyde groups, resulting in the formation of dispersions for tissue engineering. However, as confirmed by FTIR spectroscopy, the incorporation of CaO_2_ into the HA matrix did not cause oxidative cleavage of the HA.

Our study, furthermore, revealed that the novel O_2_-releasing HA-based dispersion is not cytotoxic and shows good biocompatibility. Peroxides in high concentrations, such as 35% hydrogen peroxide (H_2_O_2_), are known to cause oxidative stress and promote gingival tissue inflammation and damage in vivo [46]. In vitro, H_2_O_2_ was reported to induce senescence in different cell lines [47]. H_2_O_2_ exerted oxidative injury to primary human osteoblasts [48] and has been reported to induce senescence due to an increase of ROS in HUVECs [49] and in fibroblasts [47]. Nevertheless, the oxidative injury depends on the concentration of H_2_O_2_ and other peroxides seem to be more biocompatible, such as CaO_2_ [43]. To ensure biocompatibility of our biomaterial enclosing CaO_2_, cell viability was tested with different cell lines present in the periodontal pocket. At a concentration of 256 mg/L—a concentration creating a sufficient amount of oxygen in the dispersion—no cytotoxicity was observed against fibroblasts, osteoblasts or HUVECs. Regarding fibroblasts and HUVECs, the combination of CaO_2_ enclosed in HA increased the cell viability compared to HA respectively CaO_2._ Furthermore, a concentration of up to 512 mg/L CaO_2_ was shown to be biocompatible and induce angiogenesis in the CAM assay.

The oxygen release not only affects periodontal tissues but also increases the amount of locally available oxygen. Thus, it impairs the ecologic niche of the periodontitis-associated anaerobic bacteria and blocks their growth [50]. Therefore, oxygen-generating biomaterials may be capable of disrupting the circulus vitiosus of chronic inflammation, resulting in a hypoxic environment coupled with the overgrowth anaerobic bacteria, resulting in more inflammation.

Novel approaches that constitute an adjunct to nonsurgical periodontal therapy involve the development of selective antimicrobial agents, such as oxygen or plant extracts or probiotics, parabiotics, and postbiotics to support eubiosis [12,13,14,15,16,51]. Furthermore, host modulation is an effective adjunctive therapy and the combination of host modulation and the recovery of oral eubiosis is key to the development of targeted microbial peptides, antimicrobial peptides, and inhibitors of inflammasomes [52,53].

Taken together, the findings of this study suggest that O_2_-releasing dispersions are promising materials for the topical adjuvant therapy of periodontitis, such as a sustained oxygen release without any cytotoxic side effects. Further studies are needed to evaluate the tissue regenerating capacity of O_2_ -releasing HA.

## 4. Materials and Methods

### 4.1. Preparation of O_2_-Releasing HA-Based Dispersion

An amount of 2.56 mg CaO_2_ (200 mesh, Sigma Aldrich, St. Louis, MO, USA) was weighed into a sealable vessel. Subsequently, phosphate-buffered saline (PBS) was added, and the mixture was shaken vigorously. The desired amount of HA sodium salt (HERRLAN-PSM e.K., Alpen, Germany) was added during vigorous stirring. An amount of 300 mg of sodium hyaluronate (HA15M, Lifecore Biomedical Inc., Chaska, MN, USA) was slowly added under continuous stirring. The mixture was allowed to dissolve overnight under continuous stirring.

### 4.2. Energy-Dispersive X-ray Analysis (EDX)

EDX measurements were performed using a Hitachi SU 8000 microscope coupled with a Bruker XFlash 5010 detector. This combination of devices was applied at 8 kV, in order to acquire an elemental map of the deposits on the surfaces. Pt sputtering (7 nm) of the air-dried sample was used to avoid charging artifacts due to high probe currents needed for EDS.

### 4.3. Fourier-Transform Infrared Spectroscopy (FTIR)

The infrared (IR) spectra of lyophilized samples were recorded by a Tensor 27 FTIR spectrometer equipped with a diamond ATR unit and are reported in terms of frequency of transmission. The data were collected in a spectral range of 4000–400 cm^−1^, 16 scans, and a resolution of 4 cm^−1^.

### 4.4. Oxygen Release Measurements

An amount of 50 mg CaO_2_ (75% 200 mesh, Sigma Aldrich) was suspended in 5 mL deionized water (millipore grade). An amount of 115 mg of hyaluronic acid sodium salt (1.5–2.5 MDa: Herrlan-PSM e.K., Alpen, Nordrhein-Westfalen, Germany) was added under vigorous stirring. The mixture was stirred for at least 4 h and then inserted into a syringe without bubbles. The concentration profile of dissolved oxygen was determined by means of a benchtop dissolved oxygen meter HI 5421 (Hanna Instruments, Vöhringen, Germany). The sensor was placed in a thermostated beaker immediately above a magnetic stirring bar. The beaker was filled with 200 mL of 0.1 M citric acid (≥99.5%: Carl Roth, Karlsruhe, Germany) in 0.9 wt% NaCl aqueous solution. The pH was adjusted by means of 1 M NaOH (Carl Roth). A cellulose filter bag was placed into the solution to enable free migration of dissolved substance and prevent the free migration of the hyaluronic acid matrix or undissolved CaO_2_ particles. A baseline reading was acquired in the solution in the absence of the oxygen-releasing material for 50 min. Then, 5 mL of the test solution were added into the cellulose filter bag within 30 s. The concentration profile of the dissolved oxygen was detected for several hours at baseline and with 50 mg CaO_2_ in 5 mL water, either with or without hyaluronic acid sodium salt. The concentration of the dissolved oxygen was normalized to the concentration of the dissolved oxygen at the time when samples were added.

### 4.5. Cell Isolation

Primary human cells were obtained from patients who underwent surgery at the University Medical Center Mainz, Germany. The study was conducted in accordance with the Declaration of Helsinki and approved by the Institutional Review Board of the University Medical Center of the Johannes Gutenberg University, Mainz, Germany, according to the general terms and conditions, §14 “further use of human material” of the contract of the University Medical Center Mainz. All patients provided written consent.

#### 4.5.1. Fibroblasts

Fibroblasts were obtained from human oral mucosa. Tissue samples were cut into small pieces of approximately 2 × 2 mm with a sterile disposable scalpel. Prior to cell isolation, the tissue pieces were stepwise sterilized in 70% ethanol, in sterillium^®^ classic pure (Bode Chemie GmbH, Hamburg, Germany), and again in 70% ethanol. Then, they were transferred to 5–10 mL (depending on the amount of tissue) 0.5% protease solution (P6141, Sigma-Aldrich, St. Louis, MO, USA) in phosphate-buffered saline (PBS; Sigma-Aldrich, St. Louis, MO, USA) and incubated overnight at 4 °C. The next day, the protease solution was incubated for further 15 min at 37 °C with shaking. The sample was then passed through a cell sieve (EASYstrainerTM 70 µm sterile, Greiner bio-one, Kremsmünster, Austria) with the help of a cell scraper (Falcon^®^, Corning Inc., Corning, NY, USA). Cells were pelleted by centrifugation (1500 rpm, 5 min), transferred to cell culture medium, and seeded into small cell culture flasks with a grow area of 25 cm^2^. Cells were characterized morphologically and were used at most until passage 10 to ensure primary identity. Cells were maintained in DMEM/Ham’s F12 (Gibco, ThermoFisher Scientific, Waltham, MA, USA) supplemented with 10% fetal calf serum and antibiotics (10,000 U/mL penicillin and 10 mg/mL streptomycin; Sigma-Aldrich, St. Louis, MO, USA) at 37 °C in 5% CO_2_.

#### 4.5.2. Osteoblasts

Primary human osteoblasts were isolated according to the following protocol. Human bone specimens were obtained during hip or knee joint replacement surgeries. Cancellous bone fragments were removed with bone rongeurs from bone specimens. The isolated fragments were washed several times with PBS (Sigma-Aldrich, St. Louis, MO, USA) until a clear supernatant was achieved. The supernatant was discarded and 15 mL collagenase type I solution (1 mg/mL in medium 199, Gibco, ThermoFisher Scientific, Waltham, MA, USA) was added. Collagenase digestion was carried out under mechanical stirring in a water bath at 37 °C. After 45 min, the fragments were washed again several times with PBS (Sigma-Aldrich, St. Louis, MO, USA). The washed bone pieces were transferred into 6-well tissue culture plates with sterile forceps, followed by addition of DMEM/F-12 medium supplemented with 20% fetal calf serum (FCS) and 1% penicillin/streptomycin (PS). After the first passage, human osteoblasts were cultured in DMEM/Ham’s F12 (Gibco, ThermoFisher Scientific, Waltham, MA, USA) supplemented with 10% fetal calf serum and antibiotics (10,000 U/mL penicillin and 10 mg/mL streptomycin; Sigma-Aldrich, St. Louis, MO, USA). The medium was changed twice a week. For osteoblast differentiation, the medium was supplemented with 10 nM dexamethasone, 3.5 mM b-glycerophosphate, and 50 μg/mL ascorbic acid.

#### 4.5.3. Primary Human Umbilical Vein Endothelial Cells (HUVEC)

Primary human umbilical vein endothelial cells (HUVEC) were isolated from the vein of the umbilical cord. The umbilical cord was flushed with PBS (Sigma-Aldrich, St. Louis, MO, USA) until the buffer was clear and blood clots in the vein were removed. Then, collagenase was injected into the umbilical cord, and it was placed in PBS and incubated for 12 min at 37 °C. After incubation, the collagenase solution containing endothelial cells was flushed from the cord perfusing the vein with PBS (Sigma-Aldrich). The effluent was collected and centrifuged for 10 min at room temperature with max. 420× *g*. Cells were kept in culture dishes coated with 3 mL 0.1% gelatine at 37 °C and supplemented with EBM (PromoCell, Heidelberg, Germany) with the addition of 10% fetal calf serum (FCS; PAA, Linz, Austria). Cells were incubated overnight at 37 °C and washed with PBS (Sigma-Aldrich, St. Louis, MO, USA) the next day. Endopan 3 with 3% fetal blood serum (PAN Biotech, Aidenbach, Germany) was used as culture cell medium.

### 4.6. Cellular Viability Assays

To investigate the cytocompatibility of the O_2_-releasing HA-based dispersions, the substances were added to human primary fibroblasts, osteoblasts, and human umbilical vein endothelial cells (HUVEC) in vitro and compared to untreated cells and a dead control. For this purpose, cytotoxicity tests were performed according to ISO 10993-5 by measuring the cell viability quantitatively and calorimetrically. In all tests, 30 mg/mL sodium hyaluronate and 0.256 mg/mL CaO_2_ were used. According to ISO 10993-5, cytotoxicity is defined by more than 30% reduction of the viable cells by the substance.

Cells were seeded into a 24-well plate and allowed time to adhere overnight. The cell number per well was 50,000 for fibroblasts and HUVECs and 40,000 for osteoblasts with 1.5 mL medium per well. After 24 h, cell culture medium was replaced by 400 µL fresh medium. Cells were treated with 0.256 mg/mL CaO_2_ (Sigma Aldrich, St. Louis, MO, USA), 30 mg/mL hyaluronic acid (Herrlan-PSM e.K., Alpen, Nordrhein-Westfalen, Germany), or a combination thereof. Untreated cells served as control. The substances were applied into inserts with a 10 μm-thick translucent polycarbonate membrane (Corning Inc., New York, NY, USA) with 0.4 μm pores. Those inserts then were inserted in the 24-well plate and were thus in direct contact with the cell culture medium of the cells and incubated overnight. The inserts were removed after 24 h, the cell culture medium was exchanged for 1 mL of medium with 10% AlamarBlue™ Cell Viability Reagent (ThermoFisher Scientific, Waltham, MA, USA) and the cells were incubated for 4 h at 37 °C. After 4 h, the liquid (200 µL per well) was transferred from the 24-well plate to a black 96-well plate (Greiner bio-one GmbH, Frickenhausen, Germany) for measurements. The AlamarBlue assay is based on the change of the blue color of the nonfluorescent indicator dye (resazurin) to a fluorescent pink reduced compound after acceptance of electrons. Fluorescence was measured on a Fluorescence Microplate Reader (Fluoroskan Ascent Microplate reader, ThermoFisher Scientific, Waltham, MA, USA). Results were provided as relative fluorescence using a 538 nm excitation filter and a 600 nm emission filter, normalized to untreated control.

### 4.7. Chorioallantoic Membrane Assay (CAM Assay)

Fertilized white Leghorn chicken eggs (LSL Rhein-Main GmbH, Dieburg, Germany) were incubated at 38 °C with constant humidity of 55rH in an incubator (Type 3000 digital and fully automatic, Siepmann GmbH, Herdecke, Germany). For the first three days, eggs were placed horizontally on one side to ensure that the CAM would detach from the upwards-pointing eggshell. On embryonic development day (EDD) 3, eggs were prepared by removing 5–6 mL of the albumen in order to enlarge the space between eggshell and CAM. A small window of 3 × 2 cm was cut into the upwards-pointing part of the eggshell. The window was covered with Parafilm^®^ (Sigma-Aldrich, St. Louis, MO, USA) to prevent evaporation. On EDD-8, Gelaspon Strips (Bausch & Lomb Inc.; New York, NY, USA) were cut in slices of 1 × 0.5 × 2 mm. An amount of 20 µL of each substance (512 mg/L CaO_2_, 10 mg/mL sodium hyaluronate, combination thereof) was added to the sponge. An amount of 20 µL of water was used as control. The further assessment of potential tissue adverse events was performed blinded. After 3 h, on EDD-10, and EDD-12, photographs were captured with a digital intravital fluorescence microscope (Olympus BXFM, Olympus Deutschland GmbH, Hamburg, Germany) at 100-fold magnification using the cellSens Dimension software package.

### 4.8. Broth Microdilution Assay

MIC for *P. gingivalis* (DSM No. 20709, ATCC 33277) was determined by serial microdilution as described in a previous study [54]. *P. gingivalis* was cultivated on Schaedler agar (Becton-Dickinson, Heidelberg, Germany) for 48 h under anaerobic conditions (90% N_2_, 10% CO_2_, 10% H_2_) in an anaerobic jar system (Anoxomat Mart II, Mart Microbiology BV, Lichtenvoorde, the Netherlands). An amount of 1 mL of bacterial suspensions of 1 × 10^7^ colony-forming units (0.5 McFarland standard) was added to 9 mL Wilkins Chalgrens broth (Merlin GmbH, Bornheim-Hersel, Germany). Broth microdilution was performed with a test volume of 100 μL per well using sterile 96-well plates (Greiner Bio-One GmbH, Frickenhausen, Germany). Column one served as negative control (sterile broth only), whereas column two served as positive control (bacterial suspension only). A serial dilution of 2048 mg/L CaO_2_ was performed, starting in column four. After serial dilution, 50 µL of the standardized bacterial suspension was added to each well. After an incubation period of 48 h for *P. gingivalis* at 37 °C, the plates were inspected. The MIC was determined as the lowest concentration where no visible growth was seen in the wells. Each test was repeated three times.

### 4.9. Statistical Analysis

Graphic processing and statistical analysis was performed using the GraphPad Prism software 8.4 (GraphPad Software Inc., San Diego, CA, USA). To determine whether the data were normally distributed, the Kolmogorov-Smirnov normality test was applied. If data were normally distributed, ordinary one-way ANOVA with Dunnett’s correction for multiple comparisons was used for to determine statistically significant differences of treated samples compared to the controls. If data did not match the normal distribution assumption, the Kruskal–Wallis test with Dunn’s correction for multiple comparisons was used instead. The significance level was set to *p* < 0.05 for all comparisons.

## 5. Conclusions

Taken together, our data showed that the O_2_-releasing HA was not cytotoxic for fibroblasts, osteoblasts, and HUVECS while anaerobic growth of *P. gingivalis* was inhibited, thereby demonstrating a therapeutic potential for topical oxygen-releasing biomaterials and demonstrating their potential for use in a preventive approach for the maintenance of oral eubiosis.

## 6. Patents

On 16th of February 2022, the patent “Compositions and Kits for the Prevention or Treatment of Gum Diseases” was filed at the European Patent Office in Munich and has received the official file number EP 22 157 021.1.

## Figures and Tables

**Table 1:** AUC of CaO_2_ in H_2_O compared to CaO2 dispersed in HA in H_2_O at pH 2, 6, and 8.

pH	CaO_2_ in H_2_O (mg/L × min)	CaO_2_ and HA in H_2_O (mg/L × min)
2	11.67	34.24
6	25.83	98.52
8	23.45	45.81

## Data Availability

Not applicable.

## Supplementary Materials

The supporting information can be downloaded at: https://www.mdpi.com/article/10.3390/ijms24065936/s1.
