# Supplementary material for: Oxygen-Releasing Hyaluronic Acid-Based Dispersion with Controlled Oxygen Delivery for Enhanced Periodontal Tissue Engineering
Source: Int J Mol Sci. 2023 Mar 21;24(6):5936. doi: 10.3390/ijms24065936 (PMC10059003; doi:10.3390/ijms24065936)
Supplement: Supplementary file 1 [file ijms-24-05936-s001.zip › ijms-2279667-supplementary.pdf]

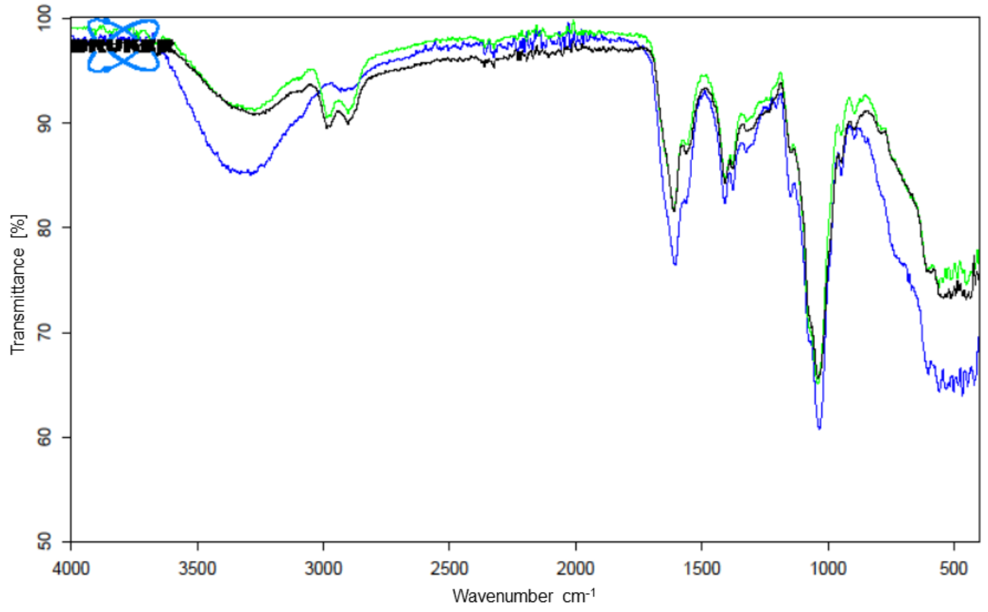

Figure S1. Comparison of IR spectra of freshly prepared mixture of PBS, HA and CaO_2 in water (black) against a sample that was stored under exclusion of ambient light for two months (green) and seven months (blue).
